# Supplementary material for: Two Congeneric Shrubs from the Atacama Desert Show Different Physiological Strategies That Improve Water Use Efficiency under a Simulated Heat Wave
Source: Plants (Basel). 2023 Jun 28;12(13):2464. doi: 10.3390/plants12132464 (PMC10347257; doi:10.3390/plants12132464)
Supplement: Supplementary file 1 [file plants-12-02464-s001.zip › plants-2440338-supplementary/Supplemental Figure 1.pdf]

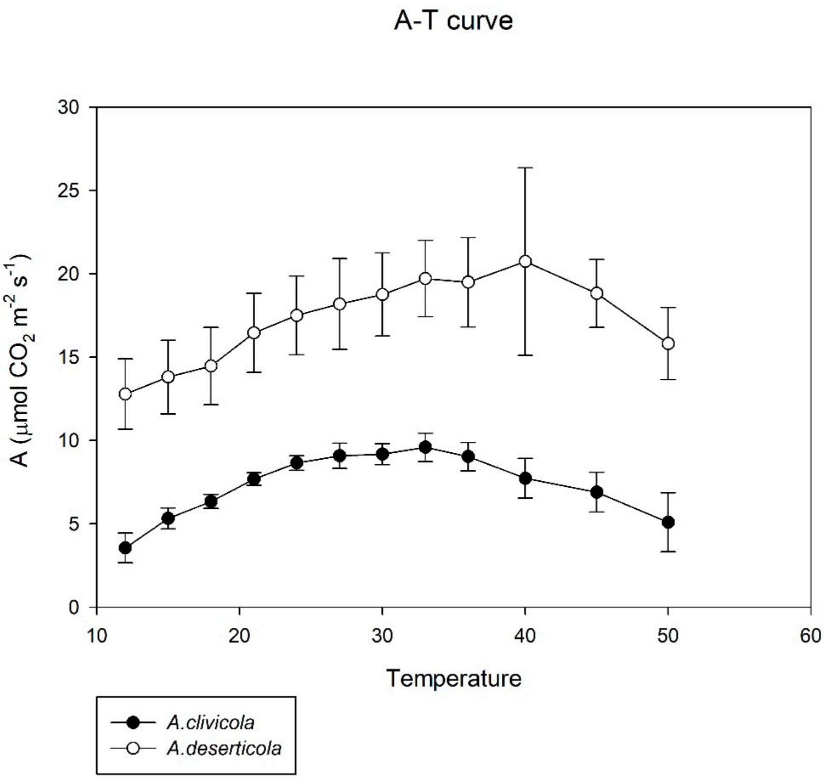

Supplemental Figure S1. Leaf temperature response curves for *A. deserticola* (open circle) and *A. clivicola* (closed circle). Vertical lines indicate standard error obtained from 5 individuals per species. See details in main text.
